# Preliminary Study on the Application of Ultrahigh Field Magnetic Resonance in Moyamoya Disease

**DOI:** 10.1155/2021/5653948

**Published:** 2021-01-13

**Authors:** Jiabin Su, Wei Ni, Baofeng Yang, Weiping Xiao, Xinjie Gao, Heng Yang, Yanjiang Li, Yu Lei, Hanqiang Jiang, He Wang, Yuxiang Gu, Ying Mao

**Affiliations:** ^1^Department of Neurosurgery, Huashan Hospital, Shanghai Medical College, Fudan University, China; ^2^Neurosurgical Institute of Fudan University, China; ^3^Shanghai Clinical Medical Center of Neurosurgery, China; ^4^Shanghai Key Laboratory of Brain Function and Restoration and Neural regeneration, China; ^5^Institute of Science and Technology for Brain-Inspired Intelligence, Fudan University, Shanghai, China; ^6^Key Laboratory of Computational Neuroscience and Brain-Inspired Intelligence (Fudan University), Ministry of Education, China; ^7^Human Phenome Institute, Fudan University, Shanghai, China

## Abstract

Magnetic resonance imaging (MRI) is widely used for the evaluation of moyamoya disease (MMD). In this paper, we describe the features of time-of-flight magnetic resonance angiography (TOF-MRA) and susceptibility-weighted imaging (SWI) at 7 T in a series of MMD patients. In this prospective pilot study, 7 patients (median age: 45.6 years; range: 30-52 years) with MMD and no contraindications for MRI underwent T2-weighted, SWI, and TOF-MRA sequences using a research 7 T head-only scanner. We show that such sequences at ultrahigh field (UHF) represent new and valuable approaches to unravel and characterize MMD. While SWI reveals more remarkable imaging signs related to an improved magnitude and phase contrast imaging, the collateral network pathways in MMD could be excellently delineated using 7 T TOF-MRA. In particular, using SWI and MRA fusion images in UHF MRI helps to improve the detection of bleeding points in hemorrhagic MMD. Our findings indicate that ultrahigh field MRI is very promising to access the severity of the disease and may facilitate revascularization surgery of MMD patients.

## 1. Introduction

Moyamoya disease (MMD) is a rare cerebrovascular pathology with a progressive steno-occlusion of the major trunks of the intracerebral arteries, accompanied with the development of collaterals called the moyamoya vessels [1]. Due to the structural and hemodynamic complexities of MMD, the radiological characteristics of this pathology have not yet been clarified. Magnetic resonance imaging (MRI) is an important tool to assess the structural, hemodynamic, and functional features of MMD [2–4]. Recent advancements in MRI acquisition, including the three-dimensional time-of-flight MR angiography (TOF-MRA) [5] and susceptibility-weighted imaging (SWI), have enabled the researchers and clinicians to elucidate the mechanisms underlying subtle changes in the vessel anatomy, brain structure, and hemodynamic status in MMD patients [3, 6, 7].

Susceptibility-weighted imaging (SWI) has been proven to have a higher lesion contrast and sensitivity, which leads to an improved detection of the cerebral hemorrhages compared with the conventional and magnitude-based techniques and/or CT [8–10]. SWI is also frequently used in the imaging of the cerebral venous vascular networks. The asymmetric cortical vessel sign (ACVS) on the SWI sequence, an asymmetric signal loss of a dilated vessel, has been reported to be an indicator for penumbra, which diminishes when hypoperfusion is ameliorated in patients with acute ischemic stroke (AIS), while the brush sign (BS) refers to the dilated deep medullary vein on the SWI sequence, reflecting an increased oxygen extraction in focal cerebral ischemia [3, 11–13].

In the past few years, the 3.0 Tesla (T) MRI has played an increasing role in the clinical practice and has been known to be an ideal tool for the comprehensive evaluation of MMD patients [13–15]. Compared with 1.5 T MRI, 3.0 T MRI showed more detailed vascular structures in moyamoya disease [16]. However, this modality still has its demerits, including the weaker delineation of small arteries, lower signal-to-noise ratio (SNR) of perfusion signals, and lower resolution of the fiber tracts [17]. The recently developed ultrahigh field (UHF) 7.0 T MRI has been put into clinical use and can provide better SNR and increased T1 relaxation, which can be utilized for the imaging of the human brain and vessel structure at a higher spatial resolution [18].

In this study, we preliminarily evaluated patients with moyamoya disease using 7 T MRI and attempted to identify some imaging features of the disease.

## 2. Methods

### 2.1. Study Population

7 individuals (4 men and 3 women; age range: 30-51years; mean age: 45.6 years) who presented with moyamoya disease from October 2019 to December 2019 underwent both 7 T MRI and digital subtraction angiography (DSA) for the diagnostic confirmation were included in this study. The study was approved by the Research Ethics Board of our institution, and signed informed consents were collected from all the participants.

### 2.2. MRI Acquisition

The acquisition protocol comprised four sequences, among which those of TOF-MRA, SWI, and T2 will be discussed in this paper. We obtained TOF-MRA, SWI, and T2 -weighted image volumes using the 7 T scanner (Siemens Terra 7 T, 32 channel head coil).  Table 1 lists the detailed acquisition parameters.

### 2.3. Digital Subtraction Angiography

All patients underwent DSA (Philips, FD2020), such that standard intra-arterial DSA was performed with femoral catheterization with 5-F catheters using a biplane DSA unit after the induction of local analgesia or general anesthesia in uncooperative patients. The standard injection rates and volumes were as follows: 4 to 5 ml/s for 8 to 10 ml for the internal carotid artery and 3 to 4 ml/s for 7 to 9 ml for the vertebral artery.

### 2.4. Interpretation and Analysis

The image processing consists of the standardized axial, coronal, and sagittal MIP images. All the postprocessed images were used by the MRI readers in their interpretation of the examination. Kendall's coefficient of concordance was calculated to determine agreement between readings (IBM SPSS Statistics 15.0).

## 3. Result

The clinical and MR imaging features of the 7 cases included in this study are shown in Table 2. The clinical and demographic data, including the age, sex, and symptoms, were acquired from the electronic medical records (EMRs). 2 patients had a history of intracranial hemorrhage, and 5 patients had a history of ischemic stroke. In total, 14 affected hemispheres were evaluated. The overall image quality was rated as excellent for most of the scans.

An independent analysis was performed by the 2 reviewing neuroradiologists and 2 reviewing neurosurgeons. Kendall's coefficient of concordance on the imaging features of the four evaluators were presented in Table 3. In the SWI sequence, all the MMD patients demonstrated the brush sign, while the ACVS was discovered in 4 of 7 patients (cases 2, 3, 4, and 6). Cerebral microbleeds were found in one case (case 7), which was the only case with clinical presentation of cerebral hemorrhage. In the TOF-MRA sequence, deeply seated collateral networks were delineated. In all the patients with intracranial hemorrhagic foci, the vessels responsible for the hemorrhage could be identified through the fusion of the MRA and SWI images.

**Figure 1:**
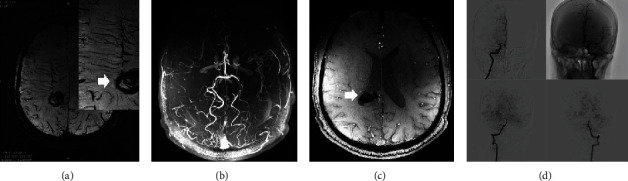
A 47-year-old male with sudden onset of slurred speech 2 months before admission and no history of intracranial hemorrhage. SWI sequence (a) illustrated that hemorrhagic foci were found in the posterior corner of the right lateral ventricle, and round low signal could be seen in the axial position of the SWI sequence. Bilateral brush signs can be clearly found in the bilateral centrum semiovale on the SWI sequence. By merging the TOF-MRA and SWI (c) sequences, it can be inferred that the cause of hemorrhage may be the rupture of the choroidal artery branch(white arrow). TOF-MRA (b) sequence showed a very high coincidence with DSA (d).

**Figure 2:**
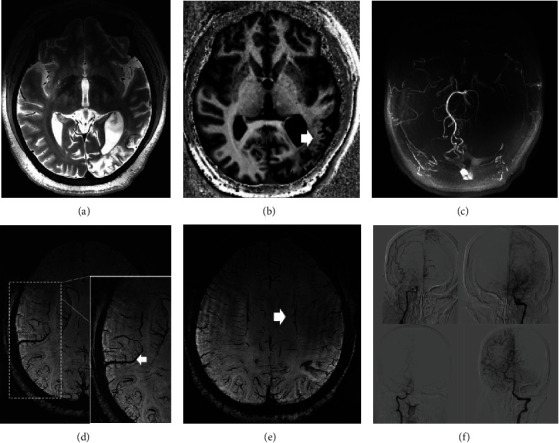
A 30-year-old female with headache and psychotic episode one month ago, T2 (a) and T1 (b) showed an infarction (white arrow) in the left temporal, parietal, and occipital lobes. TOF-MRA (c) showed an occlusion in the bilateral internal carotid artery (ICA) and left embryonic posterior cerebral artery. The SWI sequence shows the brush sign ((e) white arrow) and ACVS ((d) white arrow).

**Figure 3:**
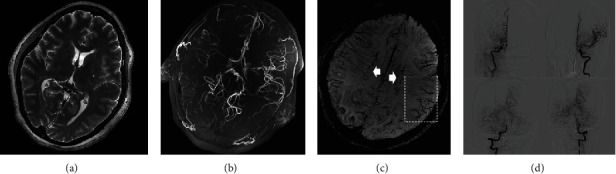
A 41-year-old female with numbness and weakness in the left limbs 2 months ago. T2 (a) showed bilateral basal ganglia lacunar infarction. TOF showed left moyamoya disease. (c) The SWI sequence illustrated brush signs (white arrows) in the bilateral centrum semiovale and ACVS in left temporal lobe (dashed box). TOF-MRA (b) sequence showed a high coincidence with DSA (d).

**Figure 4:**
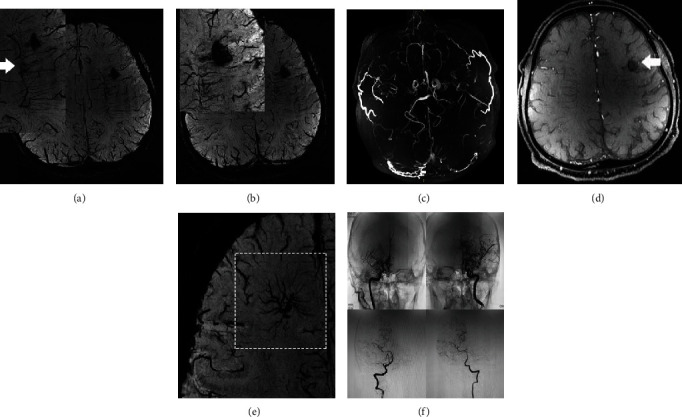
A 51-year-old male The patient suffered from sudden onset of slurred speech and right-hand weakness 8 years ago. The patient had a decline in the cognitive function in the last 2 months and denies a history of intracranial hemorrhage. (a, b) axial SWI showed low signal foci in the left frontal lobe of the SWI sequence, which was considered as a hemorrhagic lesion. In addition, the tortuous drainage vein can be seen to be dilated, and the brush sign is more obvious (c). Axial TOF showed moyamoya disease, (d) with the merge of the TOF and SWI sequences, a suspicious criminal artery was found (white arrow in (d)). ACVS was also found in this case on the SWI sequence ((e) dashed box). DSA confirmed the diagnosis of MMD and showed a high consistency with TOF-MRA.

**Figure 5:**
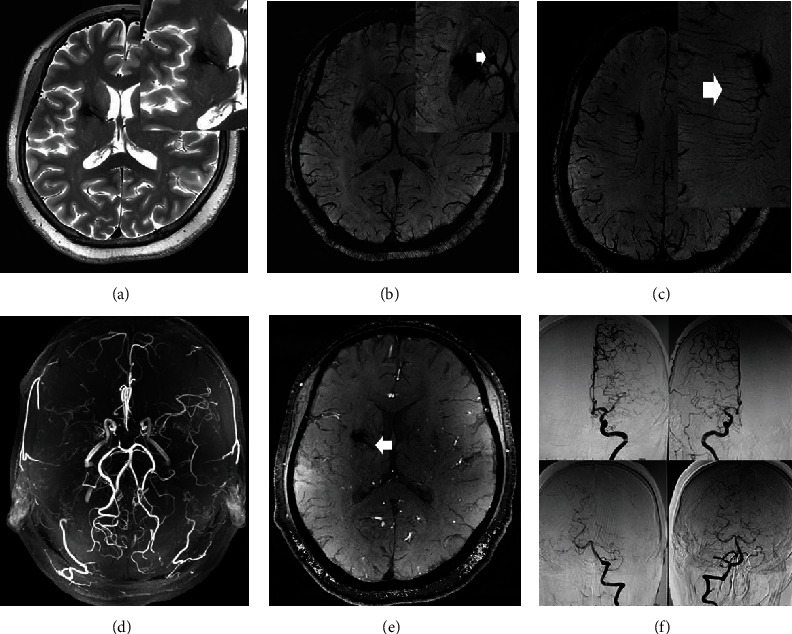
A 41-year-old female who had sudden onset of weakness in the left limbs 2 months ago. The 7 T MRI T2 sequence (a) and SWI sequence (b, c) illustrated hemorrhagic focus, venous dilatation ((b) white arrow), and brush signs. The 7 T TOF sequence (d) and the merge of TOF and SWI sequences (e) showed that the responsible bleeding artery was the lenticulostriate artery or thalamic perforator in the right basal ganglia region ((e) white arrow).

**Figure 6:**
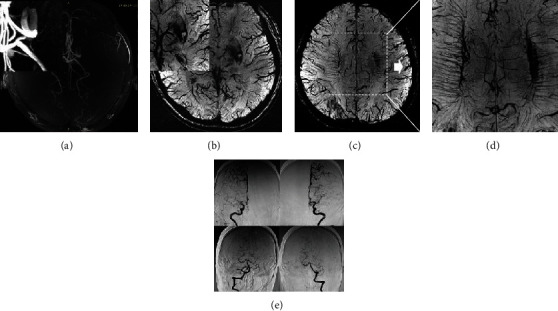
A 56-year-old male with weakness in the right limbs. The 7 T SWI sequence (b, c) showed a low signal area in the left basal ganglia region. The axial SWI sequence (d) showed an obvious brush sign in the semiovale region, especially in the left side. The SWI ((c) white arrow) sequences also showed ACVS in the left temporal parietal lobe. These findings suggested more severe ischemia in the left hemisphere.

**Figure 7:**
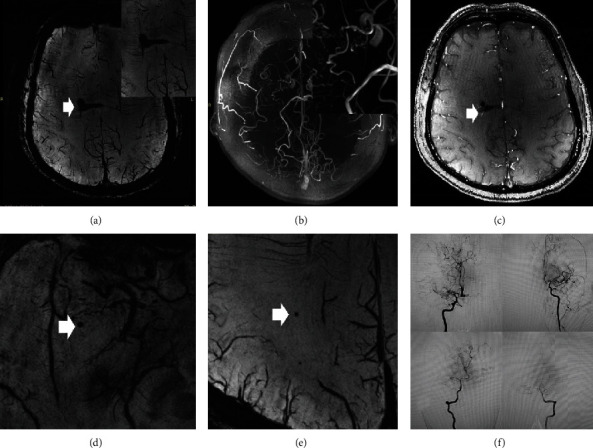
A 47-year-old male with sudden onset of severe headache. TOF-MRA and DSA demonstrated. The SWI (a) sequence illustrated right paraventricular hemorrhage. The fusion of the TOF-MRA and SWI sequences (c) showed that the choroidal artery was the most possible responsible vessel. TOF-MRA (b) demonstrated an aneurysmal structure (b). Cerebral microbleeds (CMBs) were also spotted in the SWI sequence ((d, e) white arrow).

**Figure 8:**
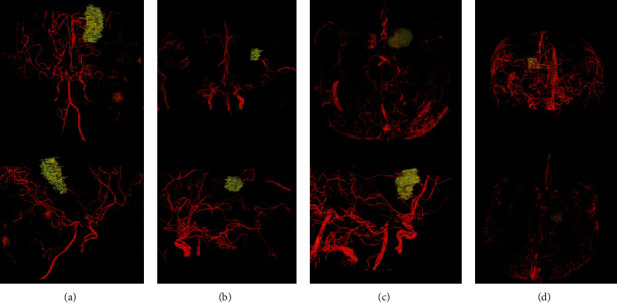
Reconstruction of TOF-MRA and hemorrhagic foci. (a) Reconstruction image of CASE1. (b) Reconstruction image of CASE 4. (c) Reconstruction image of CASE 5. (d) Reconstruction image of CASE7.

The details of the cases are as follows: case 1: 47-year-old male with asymptomatic intracranial hemorrhage (Figure 1); case 2: 30-year-old female, ischemic MMD with the brush sign and ACVS on the SWI sequence (Figure 2); case 3: 41-year-old female, ischemic MMD with the brush sign and ACVS on the SWI sequence (Figure 3); case 4: 51-year-old male with asymptomatic intracranial hemorrhage, the brush sign and ACVS were also found on the SWI sequence (Figure 4); case 5: 41-year-old female, hemorrhagic MMD (Figure 5); case 6: 56-year-old male, ischemic MMD (Figure 6); case 7: 47-year-old male, hemorrhagic MMD with cerebral microbleeds on the SWI sequence (Figure 7).  Figure 8 showed the 3D reconstruction images of MRA and bleeding foci.

## 4. Discussion

This case series of moyamoya disease patients with diverse symptoms suggests that the TOF and SWI sequences optimized for UHF are new and valuable approaches to better identify and characterize moyamoya disease.

### 4.1. TOF-MRA

In MRI, the signal-to-noise ratio (SNR) increased proportionally with the magnetic field strength and represented the most important factor to decide on the resolution. The clinical applications of TOF angiography have been somewhat limited due to the low spatial resolution obtained at 1.5 T and 3.0 T [16]. Therefore, DSA remains the criterion standard angiographic technique. In this study, the resolution of TOF-MRA is almost comparable to DSA and the detectability of the arterial structures using 7.0 T TOF could be useful in noninvasive characterization of MMD. The advantages of TOF at 7.0 T include the increased SNR, longer T1 relaxation times augmenting the vessel-tissue contrast, and inherently hyperintense arterial vasculature at higher field strengths [19–21]. Specifically, on the small intracranial vessel of TOF-MRA, 7.0 T was shown to be sensitive to the slow-flowing blood within smaller peripheral vessels when using appropriate acquisition parameters [22], which makes it a powerful tool to diagnose MMD.

### 4.2. SWI

The elevated concentration of deoxyhemoglobin in the ischemic brain tissue can be detected on SWI due to the increased blood-oxygen-level-dependent (BOLD) effects, which makes the SWI technique a good candidate to reveal the increased oxygen extraction fraction. This phenomenon may appear as hypointensity of the cortical vessels, deep white matter vessel (brush sign), and parenchyma (ischemic tissue sign).

In this case series, with a higher spatial resolution, ACVS was frequently discovered on the SWI sequence (cases 2, 3, 4, and 6). Recently, Qian and colleagues [12] observed more severely impaired hemodynamics in regions of interest (ROIs) showing ACVS, and several studies indicated that ACVS may help to evaluate the extensive ischemia in patients with acute ischemic stroke on SWI [23]. Another study reported that ACVS might be a predictor for early neurologic deterioration and unfavorable prognosis in patients with middle cerebral artery territory infarction [24]. In MMD patients, ACVS on SWI indicates a severe impairment in the hemodynamics and might be considered as a neuroimaging marker to evaluate the hemodynamics. This phenomenon may provide a basis for the choice of surgical solution for MMD. To the best of our knowledge, this is the first study reporting ACVS in MMD using the SWI sequence in 7 T MRI. In MMD, the increased conspicuity of deep medullar veins, known as the “brush sign,” was found in patients with transient ischemic attack (TIA) and infarction, implying that an impaired perfusion occurred in the deep white matter [6]. A previous study revealed that the presence of the brush sign was significantly associated with a poor outcome in patients with ischemic stroke [25]. With UHF MRI, the conspicuity of deep medullary vein increased, which enabled it to sensitively anticipate the extent of the impaired perfusion in MMD without the need to perform hemodynamic examinations, such as CTP, ASL, SPECT, or PET [26, 27]. The application of the SWI sequence in high-field MRI in the assessment of hemodynamics of patients with MMD still needs further research.

Cerebral microbleeds (CMBs) were also detected in one case (case 7), which were reported to represent a predictor for hemorrhage in MMD. CMBs are first and foremost a radiological construct describing small foci of blood degradation products in normal (or near-normal) brain tissue after extravasation of blood [28]. Depending on the MR imaging quality, such as 1.5 T versus 3.0 T, the incidence of CMBs in moyamoya disease is reported to be 12.9%–42% [29–31]. Higher fields of magnetic resonance are associated with higher detection rates. Due to the limitation of the sample size, the current study failed to conduct a quantitative statistical analysis of microhemorrhages. We believe that more studies are needed to further confirm the value of UHF MR in CMB research. Interestingly, in our study, asymptomatic bleeding foci were detected by the SWI sequence, which suggests that there are still some cases of hemorrhagic moyamoya disease that cannot be screened because of the insidious nature of its symptoms, and its potential bleeding risk is theoretically higher than that of ischemic moyamoya disease; thus, screening these patients will help to better assess the risk of intracranial hemorrhage. Additionally, asymptomatic hemorrhages in patients with MMD were predominantly observed in the periventricular region and the basal ganglia/thalami. A recent study [32] indicated that microbleeds reflect fragile microvessels with an altered blood-brain barrier (BBB), and the microbleeds are the result of blood product extravasation. Long-term hemodynamic overstress is thought to induce a rupture/extravasation of the dilated, fragile moyamoya vessels. In this case series, with the high resolution of UHF MRI, all the three different stages of hemorrhagic moyamoya disease could be observed: CMBs, asymptomatic hemorrhagic foci, and intracranial hemorrhage. We believe that the early detection of CMBs, as well as asymptomatic hemorrhagic foci and early revascularization, may prevent the progression to symptomatic or even fatal intracranial hemorrhage.

Miyakoshi and his colleagues [11] were the first to propose the identification of the bleeding point in hemorrhagic moyamoya disease using fusion images of susceptibility-weighted imaging and time-of-flight MRA. However, in the same study, the bleeding point could not be detected on SWI or TOF-MRA fusion images in around 20%–30% of the cases. In our case series, the responsible vessels were spotted in all the patients with hemorrhagic foci in the fusion images. UHF MRI makes this technique more reliable in finding the responsible arteries and assessing the risk of rebleeding. Revascularization surgery was thought to be effective to reduce the risk of rebleeding [33, 34], and this technique will provide an objective and sensitive imaging marker for the effectiveness of the surgery.

Several limitations of this study should be addressed. First, both ACVS and BS are subjective evaluations that are challenging to quantify. Second, the dynamic follow-up of these imaging features was not conducted in this study, and further longitudinal studies are needed to determine whether these features progress along with the disease and disappear after the surgical intervention.

## 5. Conclusion

This case series showed that the UHF SWI sequence improved the characterization of already known lesions, which emphasizes the delicate intricacy of cortical and vascular anatomy at the subcentimetric level and represents a promising approach to further increase the sensitivity to MMD. In conclusion, MRI at UHF may be promising for the evaluating of MMD especially the risk of hemorrhage.

## Figures and Tables

**Table 1:** Magnetic resonance sequence parameter.

	T2	TOF-MRA	SWI
Sequence	PDw/T2W	fl_tof_tra_p3_highRes	SWI
TR (ms)	6510	20	21
TE (ms)	49	3.54	14
Flip angle	135°	22°	10°
FOV (mm^2^)	210∗210	181∗200	178∗220
Voxel size(mm2)	0.5∗0.37∗2	0.5∗0.4∗0.42	0.5∗0.5∗0.75
Band width	240 Hz/Px	185 Hz/Px	205 Hz/Px
Slice thickness (mm)	2.5	0.4	0.7

**Table 2:** Magnetic resonance imaging signs and clinical features of the case series.

Case no./age (yr)/sex	Symptoms	Suzuki stage	Brush sign	ACVS	Operation	Postoperative complication	MRS
1/47/M	Sudden onset of slurred speech 2 months before admission	III-III	Yes	No	Right STA-MCA bypass+EDMS	No	0
2/30/F	Headache and psychotic episode one month before admission	V-V	Yes	Yes	Left EDMS	Epilepsy	2
3/49/F	Weakness in left limbs for 2 months	II-III	Yes	No	Conservative treatment	/	0
4/51/M	Slurred speech and right hand weakness for 8 years	IV-IV	Yes	Yes	Left STA-MCA bypass+EDMS	No	0
5/45/F	Sudden onset weakness in right limbs for 2 months	III-III	Yes	No	Right STA-MCA bypass+EDMS	Transient neurological dysfunction	1
6/45/M	Weakness in right limbs for 3 years	III-III	Yes	Yes	Conservative treatment	/	1
7/52/M	Patient with sudden onset of severe headache	III-III	Yes	No	Conservative treatment	/	0

MRS: Modified Rankin Scale.

**Table 3:** The Kendall's coefficient of concordance on the imaging features of the four evaluators.

	Kendall's WA	*P*
Brush sign	0.562	0.036^∗^
ACVS	0.674	0.013^∗^
Hemorrhage	0.906	0.001^∗∗^
MB	0.703	0.01^∗^

## Data Availability

The clinical data used to support the findings of this study are available from the corresponding author upon request.
